# Development and validation of an LC-MS/MS method for detection and quantification of *in vivo* derived metabolites of [Pyr^1^]apelin-13 in humans

**DOI:** 10.1038/s41598-019-56157-9

**Published:** 2019-12-27

**Authors:** Duuamene Nyimanu, Richard G. Kay, Petra Sulentic, Rhoda E. Kuc, Philip Ambery, Lutz Jermutus, Frank Reimann, Fiona M. Gribble, Joseph Cheriyan, Janet J. Maguire, Anthony P. Davenport

**Affiliations:** 1 Experimental Medicine and Immunotherapeutics, University of Cambridge, Level 6, Centre for Clinical Investigation, Box 110, Addenbrooke’s Hospital, Cambridge, CB2 0QQ UK; 2 Late-stage Development, Cardiovascular, Renal and Metabolism (CVRM), BioPharmaceuticals R&D, AstraZeneca, Gothenburg, Sweden; 3 Research and Early Development, Cardiovascular, Renal and Metabolism (CVRM), BioPharmaceuticals R&D, AstraZeneca, Cambridge, UK; 4 Metabolic Research Laboratories, Institute of Metabolic Sciences, University of Cambridge, Addenbrooke’s Hospital, Cambridge, CB2 0QQ UK

**Keywords:** Mass spectrometry, Translational research

## Abstract

[Pyr^1^]apelin-13 is the predominant apelin peptide isoform in the human cardiovascular system and plasma. To date, few studies have investigated [Pyr^1^]apelin-13 metabolism *in vivo* in rats with no studies examining its stability in humans. We therefore aimed to develop an LC-MS/MS method for detection and quantification of intact [Pyr^1^]apelin-13 and have used this method to identify the metabolites generated *in vivo* in humans. [Pyr^1^]apelin-13 (135 nmol/min) was infused into six healthy human volunteers for 120 minutes and blood collected at time 0 and 120 minutes after infusion. Plasma was extracted in the presence of guanidine hydrochloride and analysed by LC-MS/MS. Here we report a highly sensitive, robust and reproducible method for quantification of intact [Pyr^1^]apelin-13 and its metabolites in human plasma. Using this method, we showed that the circulating concentration of intact peptide was 58.3 ± 10.5 ng/ml after 120 minutes infusion. We demonstrated for the first time that in humans, [Pyr^1^]apelin-13 was cleaved from both termini but the C-terminal was more susceptible to cleavage. Consequently, of the metabolites identified, [Pyr^1^]apelin-13_(1–12)_, [Pyr^1^]apelin-13_(1–10)_ and [Pyr^1^]apelin-13_(1–6)_ were the most abundant. These data suggest that apelin peptides designed for use as cardiovascular therapeutics, should include modifications that minimise C-terminal cleavage.

## Introduction

Apelin is an endogenous ligand of the apelin receptor, initially characterised from bovine stomach extracts as a 77-amino acid preproprotein^1^. The prepro-apelin is further cleaved into shorter but functional fragments including apelin-36, apelin-17, apelin-13 and [Pyr^1^]apelin-13 that contain an evolutionary conserved 12-amino acid C-terminal^1–3^. [Pyr^1^]apelin-13 was subsequently identified as the most predominant isoform of the apelin family of peptides in the cardiovascular system^4,5^, and the major circulating form of the peptide^6^.

In the cardiovascular system, apelin is the most potent endogenous inotropic agent yet identified^4^. Apelin modulates vascular tone *in vivo*, decreasing blood pressure when infused into rats and dilating resistance vessels when infused into human forearm^7–9^. *In vitro*, apelin causes nitric oxide-dependent vasodilation of human splanchnic artery^10^, although a nitric oxide-independent, prostanoid dependent vasodilation in humans has been reported^4^. Apelin acted as a vasoconstrictor in endothelium denuded vessels via a direct action on vascular smooth muscle cells whilst also acting as a potent angiogenic factor and mitogen of endothelial cells^4,11^. Based on these beneficial effects, apelin was proposed as a potential therapeutic target in cardiovascular diseases. For example, apelin administration showed cardioprotective effects in heart failure^12^, and ameliorated the development of pulmonary arterial hypertension in rats^13,14^ and humans^15^. In addition, the protective effects of apelin has been reported in metabolic diseases where it decreased adiposity, serum insulin and increased insulin sensitivity^16,17^; and in renal diseases where it decreased acute renal injury and fibrosis^18^. It has recently emerged that apelin has pro-tumorigenic effects in various cancer models possibly by promoting angiogenesis and that inhibition of the apelin pathway was protective against tumour growth^14,19^. However, these beneficial effects of apelin peptides are limited by the rapid *in vivo* metabolism.

Previous studies investigating the metabolism of apelin peptides were largely conducted in plasma *in vitro* or in rodent models neither of which may represent metabolism in humans. These studies demonstrated that apelin peptides are very labile in plasma with a half-life of less than 1–5 minutes *in vitro*^20–24^. This plasma instability has to date been attributed the enzymatic activity of neprilysin^25^ and angiotensin converting enzyme II (ACE2)^22–24^, and more recently plasma kallikrein^26,27^. Similarly, another recent study reported more rapid degradation of [Pyr^1^]apelin-13 in rat and mouse plasma when compared to dog, monkey and human plasma *in vitro*^28^. The authors also confirmed their findings *in vivo* in rat and mouse, and identified N-terminal metabolites of the peptide, particularly apelin-7 that was most abundant^28^. This study therefore highlighted species differences in the repertoire of proteases circulating and present in rodent and higher mammalian systems. However, to date no studies have investigated the metabolism of apelin peptides *in vivo* in humans. The aim of this study was to develop a highly sensitive mass spectrometry based method for detection and quantification of apelin peptides in plasma. We used this method to measure intact [Pyr^1^]apelin-13 and its metabolites generated in humans, following a constant 120 minutes infusion of the peptide. We found that [Pyr^1^]apelin-13 was cleaved into smaller fragments from both termini but that the C-terminal was more susceptible. We identified the biologically active C-terminal cleavage product, [Pyr^1^]apelin-13_(1–12)_, as the most abundant, as well as identifying novel metabolites including [Pyr^1^]apelin-13_(1–10)_ and [Pyr^1^]apelin-13_(1–6)_.

## Results

### Precision and accuracy of the extraction and quantification method

**Figure 1:**
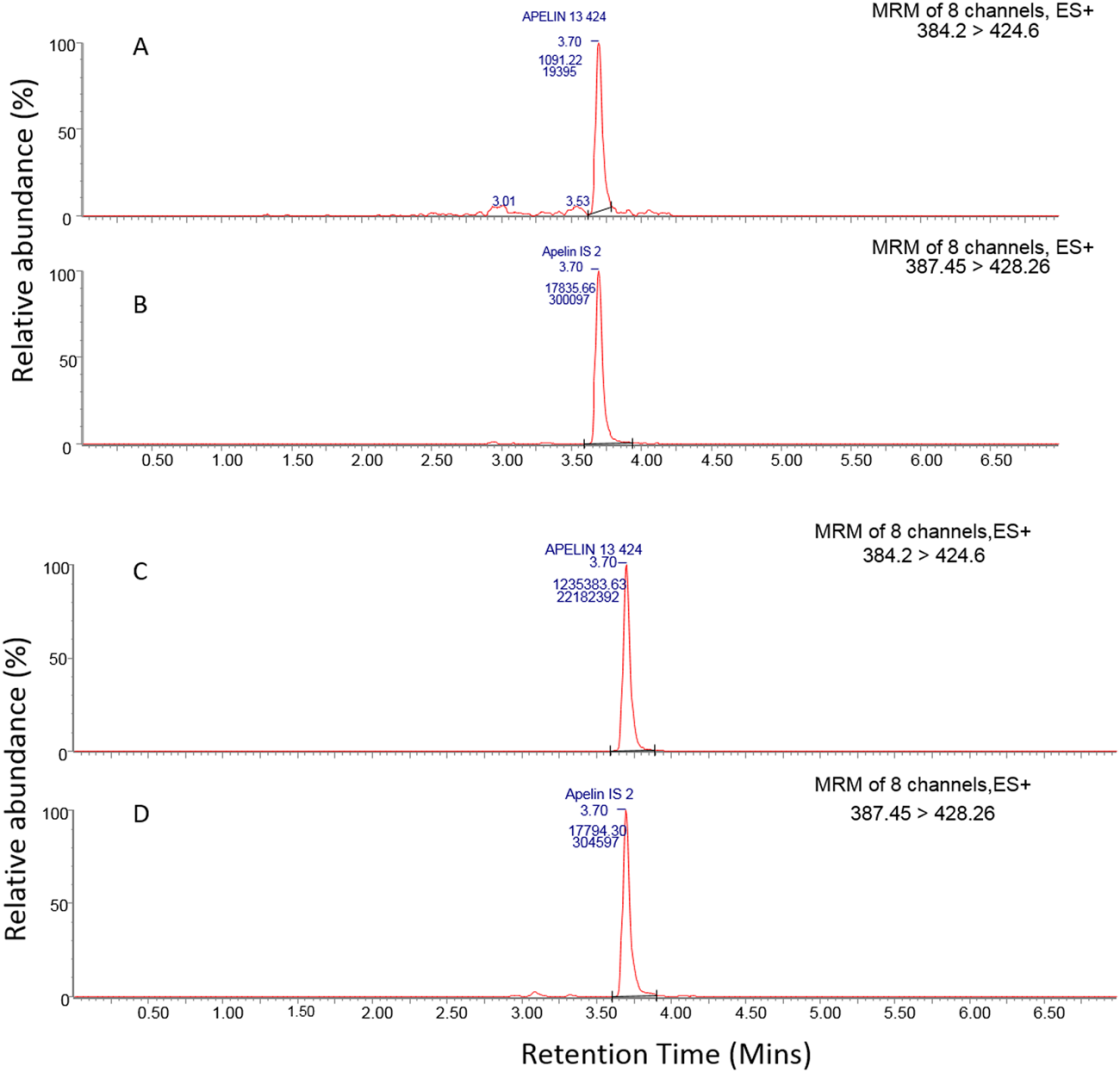
Representative chromatogram of calibration standards. LLOQ standard shows peaks corresponding to [Pyr^1^]apelin-13 at 1 ng/ml (**A**) and [Pyr^1^]apelin-13 internal standard at 25 ng/ml (**B**). Upper limit of quantification shows peaks corresponding to [Pyr^1^]apelin-13 (**C**) and [Pyr^1^]apelin-13 internal standard at 25 ng/ml (**D**). MRM = multiple reaction monitoring.

An 8-point calibration line was generated for [Pyr^1^]apelin-13 in human plasma (r^2^ = 0.99, data not shown), with a lower limit of quantification (LLOQ) of 1 ng/ml. The relative errors (% RE) for all calibration standards were less than 20% at the LLOQ and below 15% at other levels, conforming with typical bioanalytical method validation guidelines^29^. The precision and accuracy of the QC samples showed that the method was robust and accurate. The LLOQ samples returned a coefficient of variation (%CV) of 8.0 and %RE of 15.5, whilst the other QC levels had %CV’s below 6.1 and %RE’s below 8.4. Representative chromatograms obtained from calibration standards 1 and 8 are shown in Fig. 1.

### Plasma concentrations of [Pyr^1^]apelin-13 in healthy human volunteer samples

**Figure 2:**
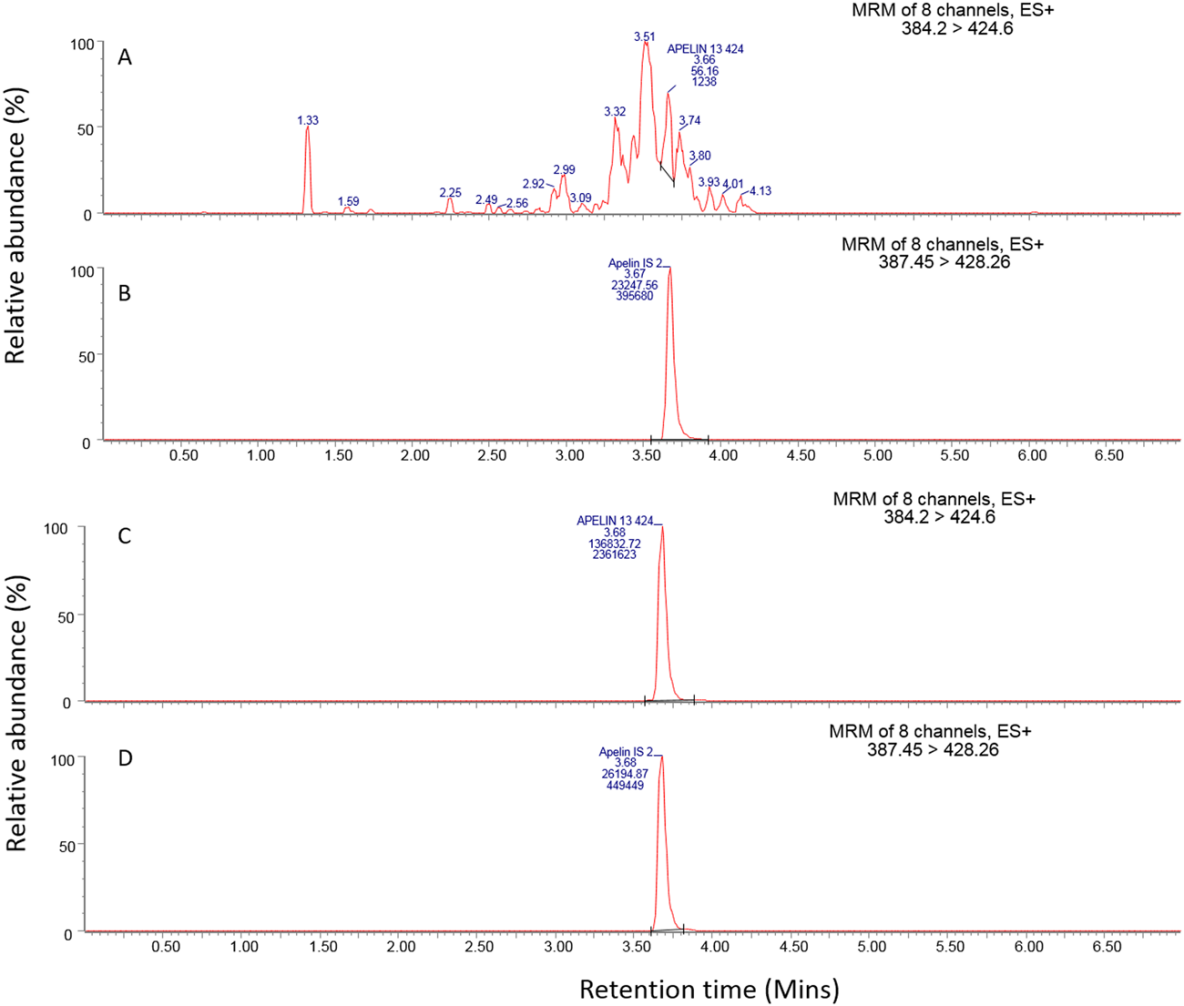
Representative chromatogram for [Pyr^1^]apelin-13 and its internal standard in volunteer samples. (**A**,**B)** chromatograms for samples obtained at t = 0 minutes; (**C**,**D**) chromatograms for samples obtained at t = 120 minutes. (**A**) no [Pyr^1^]apelin-13 was detected; (**B**) [Pyr^1^]apelin-13 internal standard chromatogram showing 3.67 minutes retention time; (**C**) [Pyr^1^]apelin-13 chromatogram showing 3.68 minutes retention time; (**D**) [Pyr^1^]apelin-13 internal standard. MRM = multiple reaction monitoring.

In samples obtained before infusion of [Pyr^1^]apelin-13, no chromatographic peak was observed for the peptide (Fig. 2A,B). Samples obtained at the end of the infusion (t = 120 minutes) showed strong peaks at 3.68 minutes corresponding to [Pyr^1^]apelin-13 (Fig. 2C,D). The measured concentration of [Pyr^1^]apelin-13 in these samples after 120 minutes was 58.3 ± 10.5 ng/ml. Additionally, data from the six donor control samples that did not receive [Pyr^1^]apelin infusion showed that the endogenous levels of [Pyr^1^]apelin in these samples were below the LLOQ (see Supplementary Fig. 1). The peak height obtained from the chromatogram of these donor samples had a maximum height that was 19.8% of that seen in the LLOQ and so was considered as blank for quantitative purposes based on the FDA method validation guidelines for demonstrating selectivity of an LC-MS methodology^30^.

### Identification of potential C-terminal metabolites of [Pyr^1^]apelin-13

**Figure 3:**
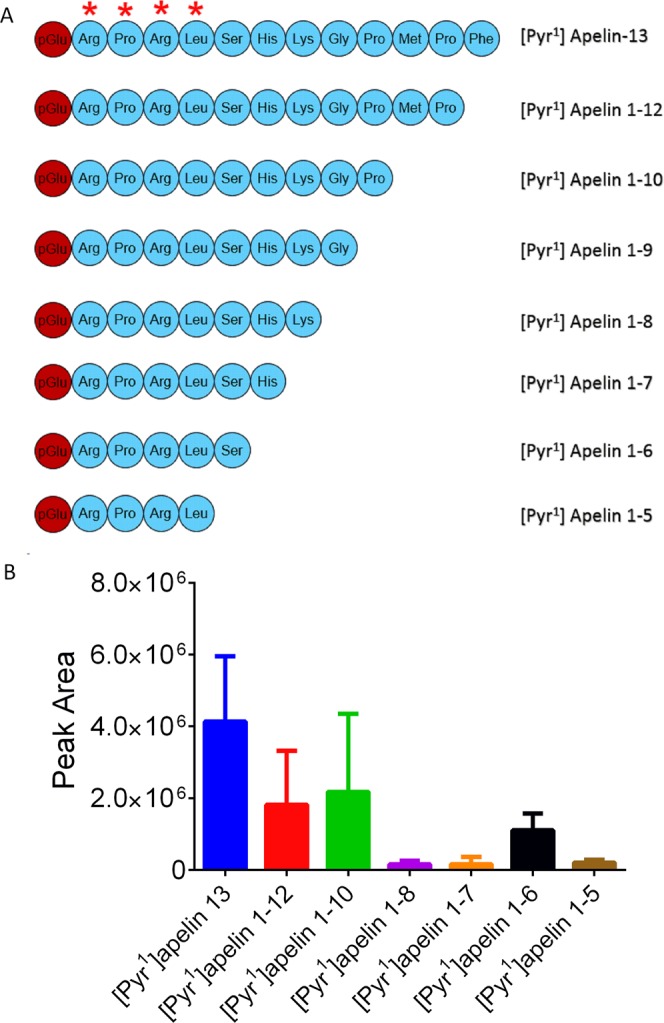
Relative abundance of [Pyr^1^]apelin-13 C-terminal metabolites identified from human plasma identified by LC-MS/MS. (**A**) peptide sequences of [Pyr^1^]apelin-13 metabolites identified in human plasma (the RPRL motif required for receptor binding was indicated by (*); (**B**) relative peak area of the metabolites (n = 6, data represent mean ± SD).

**Figure 4:**
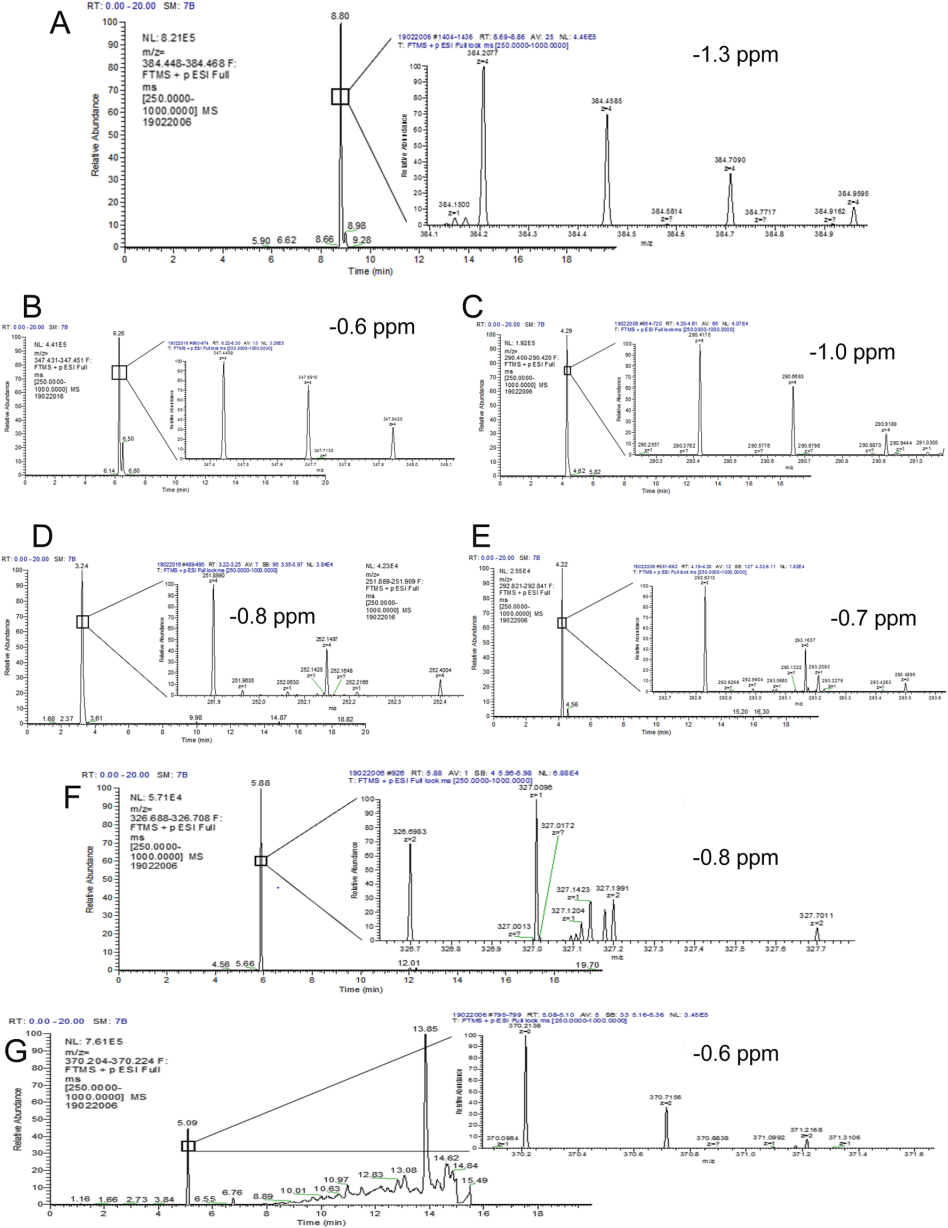
Representative chromatogram of [Pyr^1^]apelin-13 and its C-terminal metabolites identified in human plasma. The extracted ion chromatogram for [Pyr^1^]apelin-13 shows the *m/z* for the first ^13^C ion, as the extracted chromatogram for the monoisotopic peak had significant background noise throughout. (**A**) [Pyr^1^]apelin-13 with 8.80 minutes retention time; (**B**) [Pyr^1^]apelin-13_(1–12)_ with 6.26 minutes retention time; (**C**) [Pyr^1^]apelin-13_(1–10)_ with 4.29 minutes retention time; (**D**) [Pyr^1^]apelin-13_(1–8)_ with 3.25 minutes retention time; (**E**) [Pyr^1^]apelin-13_(1–7)_ with 4.22 minutes retention time; (**F**) [Pyr^1^]apelin-13_(1–6)_ with 5.09 retention time; (**G**) [Pyr^1^]apelin-13_(1–5)_ with 5.88 minutes retention time. These data were acquired using Orbitrap Mass spectrometer used for metabolite identification. The mass accuracy of the experimentally acquired monoisotopic peak was calculated for each potential metabolite, and is included along with the ^13^C isotopic cluster for each peptide with their corresponding chromatogram.

**Figure 5:**
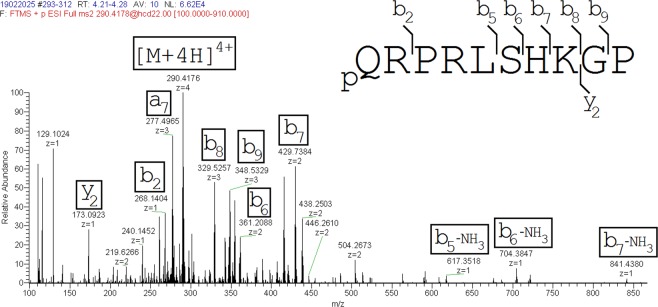
Product ion mass spectrum of [Pyr^1^]apelin-13_(1–10)_. The major ions identified are shown in brackets.

**Figure 6:**
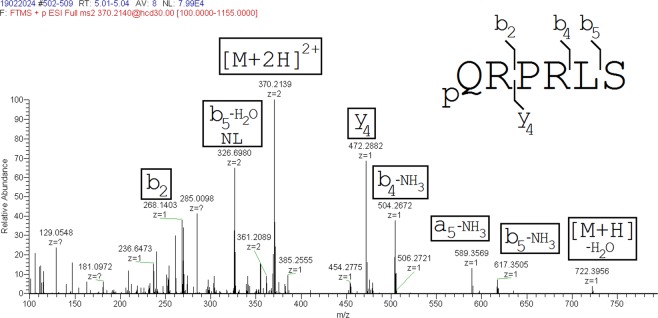
Product ion mass spectrum of [Pyr^1^]apelin-13_(1–6)_. The major ions identified are shown in brackets.

In order to identify potential metabolites of [Pyr^1^]apelin-13 generated during the 120 minutes infusion period, samples were re-analysed using a high resolution mass spectrometer. Full scan LC-MS data were interrogated for potential [Pyr^1^]apelin-13 derived metabolites by comparing extracted ion chromatograms for each analyte in the 0 and 120 minute samples in the Qualbrowser software package (Thermofisher). Peptides that were identified in the 120 minute samples were mainly generated by the loss of C-terminal amino acids (Fig. 3A). Their relative abundance in the samples are displayed in Fig. 3B. Notably, the most abundant fragments were [Pyr^1^]apelin-13_(1–12)_ (known to be biologically active^24^), [Pyr^1^]apelin-13_(1–10)_ and [Pyr^1^]apelin-13_(1–6)_. Other metabolites identified, although at lower levels (<10% of parent [Pyr^1^]apelin-13) included [Pyr^1^]apelin-13_(1–8)_, [Pyr^1^]apelin-13_(1–7)_ and [Pyr^1^]apelin-13_(1–5)_ that are likely to be biologically inactive. The chromatographic spectra corresponding to each of these metabolites are shown in Fig. 4A–G. In addition to [Pyr^1^]apelin-13_(1–12)_, the most abundant metabolite identified, [Pyr^1^]apelin-13_(1–10)_ (Fig. 5) and [Pyr^1^]apelin-13_(1–6)_ (Fig. 6) were present at a sufficient level to generate suitable product ion spectra allowing experimentally acquired fragments to be matched against theoretical fragments from the peptide sequence. The relative mass accuracy of all potential metabolites were generated and the experimental values were all within 1 ppm of expected values, whilst the mass accuracy of the parent peptide had the highest value of 1.3 ppm.

Oxidation of the methionine residue in [Pyr^1^]apelin-13 was identified, however since this modification was also observed in the extracted standards, it could not be ascertained if they occurred *in vivo* or as an artefact of the extraction process.

### Identification of potential N-terminal metabolites of [Pyr^1^]apelin-13

**Figure 7:**
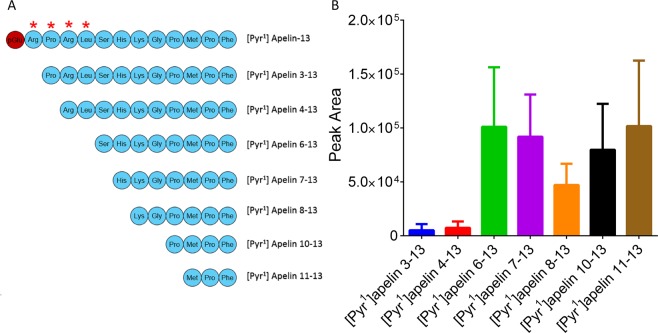
N-terminal metabolites of [Pyr^1^]apelin-13 identified from human plasma. (**A**) sequence of [Pyr^1^]apelin-13 N-terminal fragments identified, (**B**) relative abundance of N-terminal metabolites (n = 6, data represent mean ± SD). Star on the [Pyr^1^]apelin-13 residues indicate amino acid residues critical for receptor binding.

**Figure 8:**
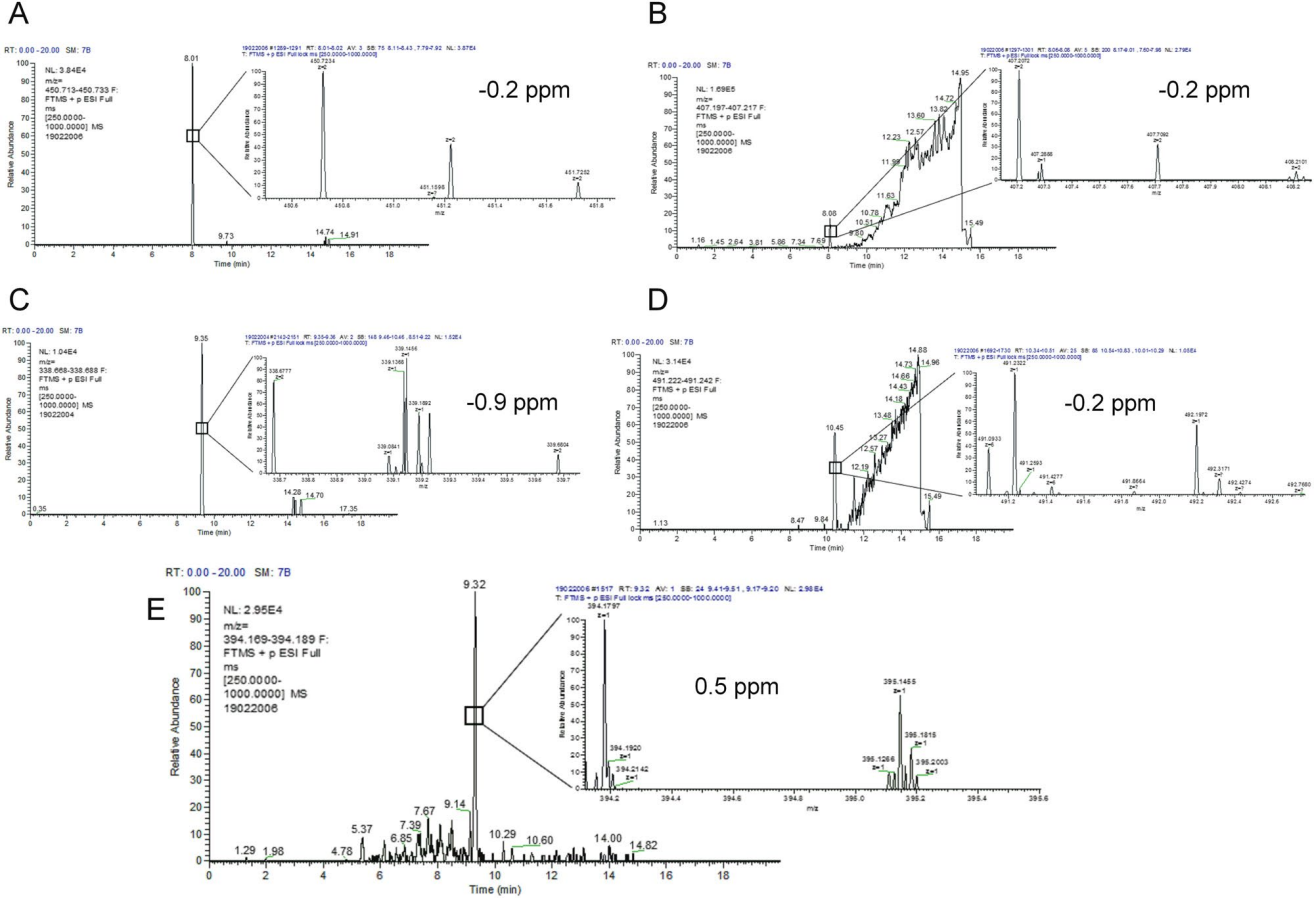
Representative chromatogram of the N-terminal metabolites of [Pyr^1^]apelin-13 identified in human plasma. (**A**) [Pyr^1^]apelin-13_(6–13)_ with retention time of 8.01 minutes, (**B**) [Pyr^1^]apelin-13_(7–13)_ with retention time of 8.08 minutes, (**C**) [Pyr^1^]apelin-13_(8–13)_ with retention time of 9.35 minutes, (**D**) [Pyr^1^]apelin-13_(10–13)_ with retention time of 10.45 minutes, (**E**) [Pyr^1^]apelin-13_(11–13)_ with retention time of 9.32 minutes. The mass accuracy of the experimentally acquired monoisotopic peak was calculated for each potential metabolite, and is included along with the ^13^C isotopic cluster for each peptide with their corresponding chromatogram.

Using the same approach described above, several N-terminal metabolites of [Pyr^1^]apelin-13 were identified (Fig. 7). Of note, the peak areas of these fragments were lower compared to those observed for the C-terminal fragments. The most abundant N-terminal fragments observed were [Pyr^1^]apelin-13_(6–13)_, [Pyr^1^]apelin-13_(11–13)_, [Pyr^1^]apelin-13_(7–13)_ and [Pyr^1^]apelin-13_(10–13)_ (Fig. 7A,B). Other fragments present but low in abundance include [Pyr^1^]apelin-13_(3–13)_, [Pyr^1^]apelin-13_(4–13)_ and [Pyr^1^]apelin-13_(8–13)_. The mass accuracy of the experimentally acquired monoisotopic *m/z* for these metabolites are shown in Fig. 8, and were all within 0.9 ppm of expected values.

## Discussion

We have developed and validated a high resolution LC-MS/MS method for detection and quantification of [Pyr^1^]apelin-13 and relative quantification of its metabolites *in vivo* in human plasma. We have shown that this method was robust, reproducible and had a high sensitivity for [Pyr^1^]apelin-13 with an LLOQ of 1 ng/ml. Using this method, we have quantified the intact peptide after constant infusion for 120 minutes into healthy volunteers and showed for the first time that in humans *in vivo*, [Pyr^1^]apelin-13 was cleaved from both the N- and C-termini, with the C-terminus being the most susceptible to proteolytic activity. The most abundant metabolite identified by this method was [Pyr^1^]apelin-13_(1–12)_ but very high levels of [Pyr^1^]apelin-13_(1–10)_ and [Pyr^1^]apelin-13_(1–6)_ were also detected, the sequences of which were confirmed using tandem mass spectrometry and manual product ion matching.

The discovery that [Pyr^1^]apelin-13 was cleaved from both ends was unexpected since to date only cleavage from the C-terminus has been described^23,24^. Our findings may therefore better explain the extremely unstable nature of apelin peptides in plasma^6,28,31^. It is worth noting that the C-terminus was more susceptible to proteolytic activity than the N-terminus, whose metabolites were present at approximately 20-fold lower levels. This may partly be explained by the pyroglutamylation of the N-terminus which may protect this region from enzymatic activity to some degree. The N-terminus of [Pyr^1^]apelin-13 also contains the RPRL motif critical for binding to the apelin receptor^32^, hence any cleavage from this direction is likely to profoundly affect the affinity of these N-terminal fragments for the receptor. A previous study showed *in vitro* that neprilysin cleaves [Pyr^1^]apelin-13 between Arg^4^ and Leu^5^ and between Leu^5^ and Ser^6^ amino acids^25^, thereby making neprilysin the first enzyme identified to date that completely inactivates the peptide. Importantly, we have now shown in this study the presence of one of these proposed neprilysin cleavage products, [Pyr1]apelin-13_(6–13)_, in humans *in vivo* with additional evidence for cleavage of the scissile bond between Leu^5^ and Ser^6^ given by the detection of the C-terminal fragment, [Pyr^1^]apelin-13_(1–5)_. To date very few studies have investigated the metabolism of peptides *in vivo* in humans. Interestingly, like [Pyr^1^]apelin-13, arginine vasopressin was also proposed to be cleaved *in vivo* from both the C- and N- termini, with carboxypeptidase and post-proline enzymes cleaving the C-terminus of arginine vasopressin, while aminopeptidases cleaved the N-terminal region^33–35^. In contrast, other *in vivo* studies of this nature identified only a single terminus cleavage of Peptide YY^36^, growth hormone-releasing hormone^37^, liraglutide, a glucagon-like peptide-1 (GLP-1) analogue^38^ and big endothelin-1^39^.

Previous *in vitro* studies in plasma, suggested that [Pyr^1^]apelin-13_(1–12)_ was a metabolite of [Pyr^1^]apelin-13 produced by the enzymatic activity of ACE2 resulting in the removal of the C-terminal phenylalanine^20,22–24,40^. However, it was unclear whether this metabolite retained biological activity at the apelin receptor. One study argued that [Pyr^1^]apelin-13_(1–12)_ had reduced biological activity compared to the native [Pyr^1^]apelin-13 as measured by its hypotensive effects in mice^23^. However, Yang *et al*.^24^ demonstrated that the ACE2 metabolite, [Pyr^1^]apelin-13_(1–12)_ contracted human saphenous vein with sub-nanomolar potency and was a potent positive inotrope in paced mouse and human heart *ex vivo*. The authors demonstrated [Pyr^1^]apelin-13_(1–12)_ was present endogenously in the endothelium of human heart and lungs, and went on to show that it was biologically active *in vivo* in humans and rodents^24^. Similarly, a previous study reported that [Pyr^1^]apelin-13 was cleaved to [Pyr^1^]apelin-13_(1–12)_ *in vivo* in rats^22^. Consistent with these studies, our work now provides clear evidence that [Pyr^1^]apelin-13_(1–12)_ is produced endogenously in human plasma *in vivo* possibly via the activity of ACE2.

[Pyr^1^]apelin-13 was also cleaved between Pro^10^ and Met^11^ and between Ser^6^ and His^7^ resulting in generation of [Pyr^1^]apelin-13_(1–10)_ and [Pyr^1^]apelin-13_(1–6)_ but the enzyme responsible for producing these metabolites remains unknown. The corresponding N-terminal fragments of these C-terminal metabolites [Pyr^1^]apelin-13_(11–13)_ and [Pyr^1^]apelin-13_(7–13)_, were also identified. These data were consistent with a previous *in vivo* study in male rats which also identified the C-terminal metabolites^22^. The authors reported on the accumulation of [Pyr^1^]apelin-13_(1–10)_ and [Pyr^1^]apelin-13_(1–6)_ signals with time as [Pyr^1^]apelin-13 and [Pyr^1^]apelin-13_(1–12)_ signals decreased, suggesting that following ACE2 cleavage of [Pyr^1^]apelin-13 to [Pyr^1^]apelin-13_(1–12)_, other unidentified enzymes subsequently cleave both [Pyr^1^]apelin-13 and [Pyr^1^]apelin-12 into [Pyr^1^]apelin-13_(1–10)_ and [Pyr^1^]apelin-13_(1–6)_. These metabolites [Pyr^1^]apelin-13_(1–10)_ and [Pyr^1^]apelin-13_(1–6)_, retained the RPRL motif required for binding^32^, although it is unclear if they retain biological activity. Taken together, these findings may suggest that there is at least some common metabolic pathways for [Pyr^1^]apelin-13 in rats and humans *in vivo*. Further studies are required to identify the specific proteases involved.

Inhibition of degradative enzymes is a well-established strategy to generate therapeutic agents. ACE2 is an important member of the renin-angiotensin system that converts angiotensin-II to angiotensin 1–7, with the latter mediating vasodilatation, anti-proliferation, anti-apoptosis and anti-fibrotic effects^41^. In addition, ACE2 has been implicated in heart failure^42,43^, diabetic nephropathy^44,45^, acute lung failure^46^, lung injury induced by the lethal avian influenza A H5N1 virus^47^, respiratory syncytial virus^48^ and severe acute respiratory syndrome (SARS)^46^. Recently, GSK developed a recombinant human ACE2, GSK2586881 for treatment of acute respiratory distress syndrome (ARDS) and showed that this molecule was well-tolerated in clinical trials^49^. Corroborating on this, apelin signalling induces ACE2 expression in failing hearts^12^, and protects against lung injury in experimental models of acute respiratory distress syndrome^50^, possibly by inhibiting the NF-κB pathway and components of the inflammasome^51^. Furthermore, apelin counteracts the effects of angiotensin-II signalling, which is negatively regulated by ACE2, suggesting that targeting ACE2 and apelin could be a potentially novel therapeutic strategy for treatment of lung injury related pathologies and heart failure.

The beneficial effects of apelin in heart failure are well characterised. Plasma apelin levels have been suggested to increase in early stages^5^ of heart failure but decrease in late stages of the disease^52–54^. In support of this, heart failure therapies such has cardiac resynchronisation therapy used to treat refractory chronic heart failure were shown to increase plasma apelin suggesting that increased apelin levels are associated with improved therapeutic benefit^54^. Apelin administration increased stroke volume and contractility in failing hearts^11^, thereby improving the performance of the failing heart. Similarly, neprilysin inhibitors have emerged as a pivotal therapeutic strategy for clinical management of heart failure due to the role of neprilysin in the degradation of vasoactive peptides including natriuretic peptides and bradykinin^55^. Indeed, neprilysin inhibitors such as sacubitril are used for clinical management of heart failure^56^. Our data may therefore suggest that an additional benefit of neprilysin inhibitors in heart failure is to reduce apelin inactivation resulting in beneficial vasodilation, increased contractility and cardiac output. Building on these findings, further studies could substitute the amino acids at the neprilysin cleavage sites in [Pyr^1^]apelin-13 with unnatural amino acids to improve its resistance to degradation. Indeed, it was recently shown that infusion of neprilysin resistant apelin-17 in an established mice model of abdominal aortic aneurysm ameliorated the adverse aortic remodelling and aneurysm formation^27^. Such a strategy was also demonstrated to significantly increase the resistance of [Pyr^1^]apelin-13 and apelin-17 to ACE2 activity^22,23^, suggesting that this could potentially be a mechanism to improve plasma stability of apelin-based therapeutics for clinical indications. We have recently published on an another approach to stabilise apelin peptides in human blood using albumin domain (AlbudAb) antibody conjugated to an apelin analogue, MM202 and showed that this peptide was resistant to degradation yet retained biological activity at the human apelin receptor *in vitro* and *in vivo*^9^. Therefore, these strategies could in the near future result in the development of the first apelin-based therapeutics for treatment of human diseases.

In conclusion, apelin peptides have protective roles in cardiovascular diseases, however, any potential therapeutic use is impaired by the poor plasma stability of the peptide. In this study, we have developed a highly sensitive method for detection and quantification of [Pyr1]apelin-13 in human plasma. For the first time in humans *in vivo* we have identified as the most abundant metabolite of [Pyr^1^]apelin-13, the ACE2 cleavage product, [Pyr^1^]apelin-13_(1–12)_ that we have previously demonstrated retains significant biological activity in addition to the putative neprilysin metabolites [Pyr^1^]apelin-13_(4–13)_ and [Pyr^1^]apelin-13_(6–13)_. Combined inhibition of ACE2 and neprilysin may be a novel strategy to enhance endogenous apelin levels in conditions in which the peptide is downregulated. Additionally, these data will inform the design of more stable apelin peptides for therapeutic use.

## Material and Method

### Materials

[Pyr^1^]apelin-13 was custom synthesised by Severn Biotech (Kidderminster, England), and analysed by mass spectrometry and purity by HPLC analysis (99.2%) dispensed under sterile conditions. Pharmacological activity of [Pyr^1^]apelin-13 was confirmed using *in vitro* and *in vivo* assays (Supplementary Figs. 2 and 3). Peptides were stored below −40 °C in a monitored freezer until use^2^. Stable isotope labelled [Pyr^1^]apelin-13 (pGlu-R-[U-^13^C_5_, ^15^N-Pro]-R-[U-^13^C_6_, ^15^N-Leu]-SHKGPMPF-acid) was custom synthesised by Cambridge Research Biochemicals (Billingham, England). Protein LoBind Eppendorf tubes (Cat No.: 0030108094) and 1 ml protein LoBind 96-well plate (Cat No.: 0030504216) were purchased from Eppendorf (Stevenage, UK), and Oasis HLB Prime µ-Elution 96-well plates were purchased from Waters (Waters, Wilmslow, UK; Cat No.: 186008052). Acetonitrile (ACN) (Cat. No.: 270717) and glacial acetic acid (Cat. No.: 33209-1L) were purchased from Sigma Aldrich (Saint Louis, USA), methanol (Cat. No.: 10675112) and 0.1% formic acid (FA) in water (%v/v) (Cat. No.: LS118-212) were purchased from Fisher Scientific (New Hampshire, USA). ACQUITY UPLC HSS T3 1.8 μm 2.1 × 50 mm column (Cat No.: 186003538) used for the LC-MS/MS analysis was obtained from Waters (Wilmslow, UK).

### Study protocol

This study was registered on Clinicaltrials.gov (NCT03449251) and carried out with ethical approval from the Yorkshire & The Humber – Sheffield Research Ethics Committee (REC reference 18/YH/0010). All participants gave written informed consent and studies adhered to the Declaration of Helsinki. Six healthy volunteers (3 male and 3 female, mean age 43.8 ± 6.9, with body mass index within the normal range of 23.0 ± 1.0) were recruited for infusion. Volunteers were fasted and were lying supine with their heads supported in a quiet, temperature controlled (23–25 °C) room for the duration of the study. Following a period of acclimatisation, the first sample of venous blood was obtained from the arm contralateral to the arm used for infusion of apelin. Vials containing [Pyr^1^]apelin-13 were allowed to warm to room temperature and diluted with physiological saline to produce stock solutions, that were then filtered using a 0.2 µm Portex flat filter (Portex, UK) before undergoing serial dilutions with 0.9% sterile saline. There was no loss of apelin following this filtration procedure. Volunteers were infused with a concentration of 135 nmol/min of [Pyr^1^]apelin-13, at a rate of 1 ml/min for 120 minutes, using a syringe pump, equipped with a 50 ml syringe and 16 gauge catheter. The second venous sample was obtained immediately after the end of the infusion. Blood samples were collected into 2.6 ml EDTA tubes, immediately put on wet ice and centrifuged for 7 minutes at ~4 °C, 4000 rpm and stored a −70 °C, prior to analysis. A previous study had used a concentration of up to 100 nmol/min for systemic infusion, where they obtained a therapeutic response in patients with pulmonary arterial hypertension and the highest dose was well tolerated^15^. The dose chosen of 135 nmol/min of [Pyr^1^]apelin-13, was slightly higher in order to identify possible metabolites. Additional control samples were obtained from 6 donors (3 male and 3 female) within a similar age group who did not receive the apelin infusion for comparison.

### [Pyr^1^]apelin-13 LC-MS/MS and SRM based detection method development

An LC-MS/MS method was developed for [Pyr^1^]apelin-13 and its stable isotope labelled [Pyr^1^]apelin-13 analogue. LC-MS/MS instrumentation used for the quantitation of [Pyr^1^]apelin-13 included a H-Class Acquity (Waters) attached to a TQ-XS triple quadrupole mass spectrometer (Waters). Peptides were separated using a 2.1 × 50 mm 1.8 mm particle HSS T3 Acquity column held at 60 °C and flowing at 350 µl/minute. Gradient starting conditions were 95% A (0.1% FA in water v/v) and 5% B (0.1% FA in ACN). Starting conditions were held for 0.2 minutes before raising to 25% B over 4 minutes. The column was flushed with 90% B for 0.8 minutes before returning to starting conditions. The total time of each analysis was 7 minutes, with the first 1.2 minutes and last 2.8 minutes diverted to waste. The source parameters used included a positive electrospray voltage of 3.0 kV, gas flow of 1000 L/hour, desolvation temperature of 600 °C and a cone voltage of 40 V.

A full scan analysis of the peptide showed that the [M + 4 H]^4+^ charge state was the predominant ion in the spectrum as previously described by Mesmin *et al*.^31^ in their LC-MS/MS analysis of [Pyr^1^]apelin-13. Therefore this was selected for fragmentation. A product ion spectrum was collected over a range of 100 to 1600 *m/z* and two ions were selected for SRM optimisation (*m/z* 424.6 and 408.55). The 424.6 ion corresponded to the b11 fragment and the 408.55 ion was derived from the loss of a methyl-sulphide group from the methionine on the b11 ion, as previously described by Mesmin *et al*.^31^. Optimal conditions for the two SRM transitions for [Pyr^1^]apelin-13 were 384.2/408.55, 384.2/424.6 with collision energy values of 14 and 12 eV respectively. The internal standard used the same collision energy but targeted transitions of 387.45/412.88 and 387.45/428.26. Peptide peak areas were integrated using the TargetLynx program associated with Masslynx V 4.2 (Waters), and peak area ratios were generated against the corresponding stable isotope-labelled internal standard peptide peak.

### Extraction of [Pyr^1^]apelin-13 from human plasma

Plasma samples were thawed on ice and 50 µl transferred into protein LoBind Eppendorf tubes containing 25 µl GuHCl (6 M). A 300 µl aliquot of 80% ACN in water (containing 25 ng/ml internal standard) was added to all plasma samples and vortexed before centrifuging at 12000 x g for 5 minutes to precipitate plasma proteins. The supernatant was transferred to a 1 ml protein LoBind 96-well plate and evaporated. Samples were reconstituted in 500 μl 0.1% FA (v/v) and loaded onto an Oasis HLB Prime µ-elution 96-well plate (Waters, Wilmslow, UK) and slowly extracted on a positive pressure manifold (Waters). The columns were washed with 200 µl of 5% methanol in water with 1% acetic acid (v/v) and eluted from the cartridge using 2 × 50 µl of 60% methanol in water with 10% acetic acid (v/v). The eluate was evaporated to dryness and reconstituted in 150 µl 0.1% FA (v/v) in water and 10 µl was injected onto the LC-MS/MS system.

### Precision and accuracy of the extraction method

Blank plasma was pre-incubated at 37 °C for at least 2 hours, to degrade any endogenous [Pyr^1^]apelin-13 and used to generate an eight point calibration line of custom synthesised [Pyr^1^]apelin-13 over a range of 1–1000 ng/ml. A 50 µl aliquot of each calibration standard (1, 2, 5, 10, 50, 100, 900, and 1000 ng/ml) was extracted using the SPE method described above. Four levels of QC were also generated (1, 3, 100 and 800 ng/ml) and extracted six times in order to assess the precision and accuracy of the method. Calibration line followed a linear fit, and 1/x^2^ weighting was applied. Recovery of the [Pyr^1^]apelin-13 from plasma was assessed by analysing spiked solution before and after extraction at a concentration of 100 ng/ml. Plasma samples from six individuals were also extracted to assess the selectivity of the LC-MS/MS method.

### Peptide Identification using high-resolution mass spectrometry

Samples were reanalysed on a high resolution mass spectrometer to identify potential metabolites from the administered [Pyr^1^]apelin-13 peptide. A full scan analysis was performed using a ThermoScientific Ultimate 3000 LC system connected to a ThermoScientific Orbitrap Q-Exactive Plus mass spectrometer. Solvents used for the separation were A: 0.1% FA in water (v/v) and B: 0.1% FA in ACN (v/v). A volume of 30 μl of extract was injected onto a HSS T3 UPLC™column (2.1 × 50 mm; Waters, Elstree, UK) held at 60 °C and with a flow rate of 300 µL/min. A starting condition of 1% B was used to capture the more hydrophilic peptide metabolites, and these were eluted using a linear gradient up to 30% B over 16 minutes. The column was washed for 2 minutes at 90% B and returned to starting conditions for 2 minutes, totalling a run time of 20 minutes. Mass spectrometry was performed using positive electrospray mode with a needle voltage of 3 kV, gas settings of 55 and 10 for sheath gas and aux gas flow rates. The temperature of the gas was set at 350 °C and the transfer capillary at 350 °C and a s‐lens value of 70 V. Full scan data were acquired over an m/z range of 250–1000, using a resolution of 70,000 and a maximum fill time of 100 ms. Acquired LC-MS data were interrogated for potential [Pyr^1^]apelin-13 metabolites by searching for all potential cleavage products from the parent peptide in the RAW data files using the Qualbrowser software package (Thermofisher). The *m/z* values for these peptides at multiple charge states are displayed in Supplementary Table 1. The potential [Pyr^1^]apelin-13 metabolites that were manually identified were subsequently characterised, where 30 μl of sample was reinjected using a targeted MS/MS analysis. The potential [Pyr^1^]apelin-13_(1–6)_ and [Pyr^1^]apelin-13_(1–10)_ peptides were targeted using precursor ion m/z values of 370.214 (collision energy of 30) and 290.417 (collision energy of 22) respectively. The MS/MS analysis involved the same LC separation, but MS/MS data were acquired at 17,500 resolution with an AGC of 1e6 ions, lowest *m/z* value of 100 and a max fill time of 200 ms.

## Supplementary information

Supplementary information for Development and validation of an LC-MS/MS method for detection and quantification of in vivo derived metabolites of [Pyr1]apelin-13 in humans

## Data Availability

All data generated or analysed during this study are included in this published article.
