# Supplementary material for: Development and validation of an LC-MS/MS method for detection and quantification of in vivo derived metabolites of [Pyr1]apelin-13 in humans
Source: Sci Rep. 2019 Dec 27;9:19934. doi: 10.1038/s41598-019-56157-9 (PMC6934825; doi:10.1038/s41598-019-56157-9)
Supplement: Supplementary file 1 — Supplementary information for Development and validation of an LC-MS/MS method for detection and quantification of in vivo derived metabolites of [Pyr1]apelin-13 in humans [file 41598_2019_56157_MOESM1_ESM.docx]

Development and validation of an LC-MS/MS method for detection and quantification of *in vivo* derived metabolites of [Pyr^1^]apelin-13 in humans

Running title: Identification of [Pyr^1^]apelin-12 by LC-MS/MS as the principal in vivo metabolite of [Pyr1]apelin-13 in human plasma

Duuamene Nyimanu^1^, Richard G. Kay^4^, Petra Sulentic^1^, Rhoda E. Kuc^1^, Philip Ambery^2^, Lutz Jermutus^3^, Frank Reimann^4^, Fiona M. Gribble^4^, Joseph Cheriyan^1^, Janet J. Maguire^1^, Anthony P. Davenport^1^

^1^Experimental Medicine and Immunotherapeutics, University of Cambridge, Level 6, Centre for Clinical Investigation, Box 110, Addenbrooke’s Hospital, Cambridge, CB2 0QQ, UK

^2^Late-stage Development, Cardiovascular, Renal and Metabolism (CVRM), BioPharmaceuticals R&D, AstraZeneca, Gothenburg, Sweden

^3^Research and Early Development, Cardiovascular, Renal and Metabolism (CVRM), BioPharmaceuticals R&D, AstraZeneca, Cambridge, UK

^4^Metabolic Research Laboratories, Institute of Metabolic Sciences, University of Cambridge, Addenbrooke’s Hospital, Cambridge, CB2 0QQ, UK

Corresponding Author Professor Anthony Davenport

Experimental Medicine and Immunotherapeutics (EMIT)

University of Cambridge, Level 6, ACCI, Box 110,

Addenbrooke’s Hospital, Cambridge, CB2 0QQ, UK

Tel +44(0)1223 336899

Fax +44(0)1223 762564

Email:apd10@medschl.cam.ac.uk

**Supplementary information**

**Methods**

*Validation of biological activity of [Pyr^1^]apelin-13 peptide used in the volunteer clinical study*

To confirm the biological activity of the custom synthesised [Pyr^1^]apelin-13 prior to infusion into the healthy volunteers, forskolin stimulated cAMP and β-arrestin recruitment assays were performed in CHO-K1 cells expressing the human apelin receptor as previously described^1,2^. Data were analysed to provide values of potency (EC_50_, the concentration of agonist producing 50% of the maximum response to that agonist; pD_2_ is the negative log_10_ EC_50_). The *in vivo* activity of the [Pyr^1^]apelin-13 was determined by intravenous bolus administration in anaesthetised rat as previously described^1–3^.

*Optimisation of extraction methods for plasma [Pyr^1^]apelin-13*

Four different conditions were used to determine the best method for extracting apelin peptides from human plasma. All sample preparation steps were done on ice to reduce *in vitro* peptide degradation. Human plasma was spiked with 100 ng/ml [Pyr^1^]apelin-13 and transferred (50 µl) to Eppendorf tubes before proteins were precipitated using 300 μl of 75%, 80%, 85% or 90% ACN in water (v/v) (condition 1) or the same ACN solution with 0.1% FA (v/v) (condition 2). In condition 3, 25 µl guanidine hydrochloride (GuHCl) was added to 50 µl plasma and mixed thoroughly before plasma proteins were precipitated with 300 µl 80% ACN in water with or without 0.1% FA (v/v). In condition 4, 450 µl water or 50 mM ammonium bicarbonate (pH 8) or 1% FA (v/v) was added to 50 µl plasma and transferred onto Oasis HLB Prime µ-Elution 96-well plates for SPE. The samples from conditions 1-3 were vortexed and centrifuged at 12000 xg for 5 minutes and the supernatant transferred to a 1 ml protein LoBind plate. Samples in condition 3 separated into two liquid layers both of which were collected. The supernatant was evaporated under a stream of oxygen-free nitrogen heated to 40 ⁰C using a Biotage SPE dry (Upsala, Sweden) evaporation system. Dried samples from conditions 2-3 were reconstituted in 200 μl 0.1% FA (v/v) and loaded unto an Oasis HLB Prime µ-elution 96-well plate (Waters, Wilmslow, UK) together with samples from condition 4 and slowly extracted on a positive pressure manifold (Waters). The columns were washed with 200 µl of 5% methanol in water with 1% acetic acid (v/v) and eluted from the cartridge using 2x 50 µl of 60% methanol in water with 10% acetic acid. The eluate was evaporated to dryness and reconstituted in 150 µl 0.1% FA in water and a volume of 15 µl was injected onto a LC-MS/MS system.

Statistical Analysis

All data were presented as mean±SEM. All cell based assay data were performed in triplicates. For the *in vivo* rat study, data obtained from [Pyr^1^]apelin-13 and saline controls were compared using two-tailed student’s *t*-test (Graphpad Prism 6) and statistical significance taken at *p˂0.05.*

**Results**

*Biological activity of [Pyr^1^]apelin-13 peptide*

[Pyr^1^]apelin-13 showed the expected potency at recruiting β-arrestin in CHO-K1 cells stably expressing the human apelin receptor following binding with an EC_50_ of 1.2 nM (supplementary figure 2A). In cAMP assay used to measure the G protein activity of the apelin pathway, [Pyr^1^]apelin-13 potently inhibited forskolin-induced cAMP release with an EC_50_ of 3.3 nM (supplementary figure 2B). We went on to test the activity of our custom synthesised [Pyr^1^]apelin-13 *in vivo* in anaesthetised rats, where we observed that [Pyr^1^]apelin-13 significantly decreased systolic pressure (supplementary figure 3A, *p<0.001*). [Pyr^1^]apelin-13 also significantly increase cardiac output (CO) and heart rate (HR) when compared to saline controls (supplementary figure 3B,C, *p<0.005 CO, p<0.05 HR*).


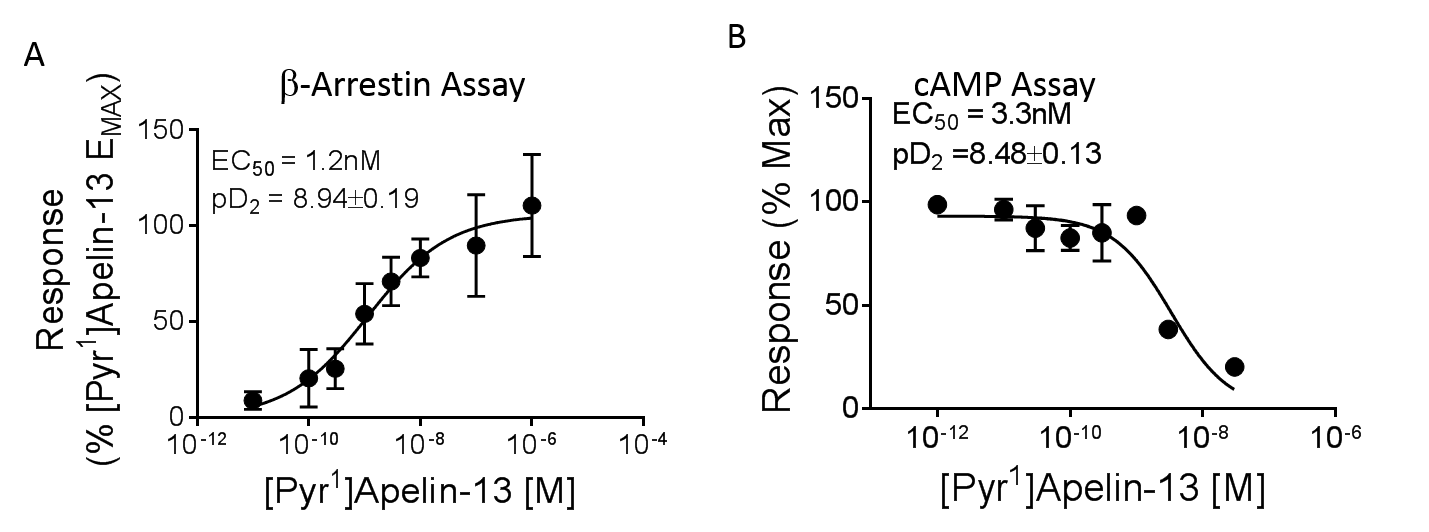


Supplementary Figure 2. The *in vitro* pharmacology of [Pyr^1^]apelin-13 in cell based β-arrestin and cAMP assays. EC_50_= the concentration of agonist producing 50% of the maximum response to that agonist; pD_2_ is the negative log_10_ of EC_50_.


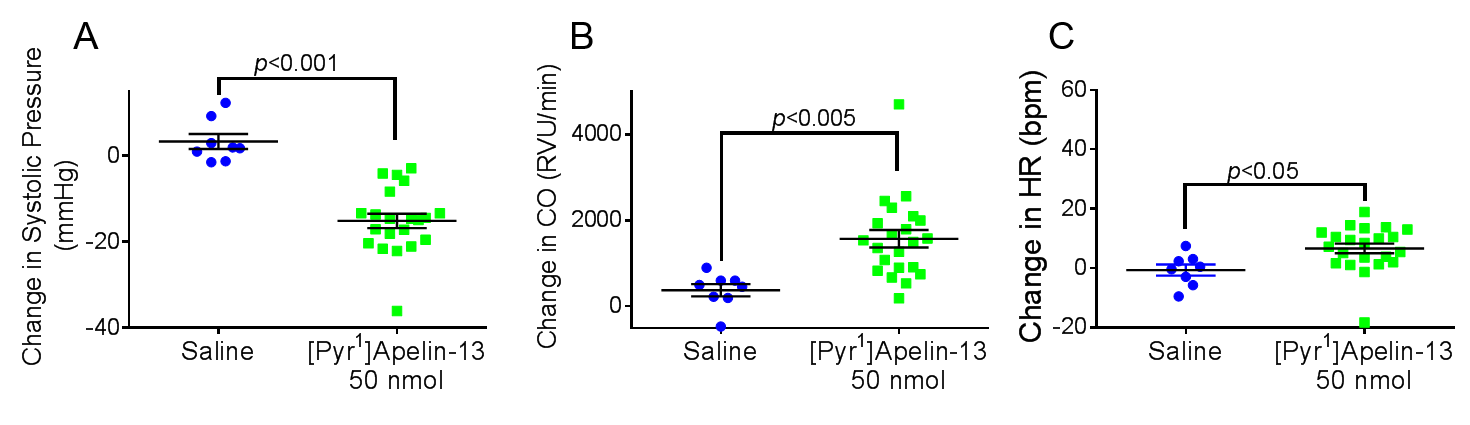


Supplementary Figure 3. The *in vivo* pharmacology of [Pyr^1^]apelin-13 in anaesthetised rats. A, effect on systolic pressure; B, effect on cardiac output (CO); C, effect on heart rate (HR). Saline treated n = 8; [Pyr^1^]apelin-13 treated n = 21, bpm = beats per minute.

*Extraction method validation*

The method for extracting apelin peptides from plasma was carefully evaluated by spiking [Pyr^1^]apelin-13 into plasma and monitoring its recovery using different extraction methods. We found that 80% ACN in water (% v/v), 75% ACN in 0.1% FA (% v/v), 75% ACN in 0.1% FA (% v/v) followed by SPE, 80% ACN in water or in 0.1% FA (v/v) plus GuHCl gave 82.0%, 99.5%, 97.7%, 95.0% and 82.0% recovery respectively (supplementary figure 4). The 80% ACN in water plus GuHCl followed by SPE resulted in slight lower recovery than the 75% ACN with 0.1% FA, however this condition was chosen for further work for two reasons. The addition of an SPE step results in cleaner extracts prior to LC-MS/MS analysis, and the addition of the GuHCl (a potent chaotrope) will disrupt peptides and potential metabolites from binding to albumin and other proteins in plasma, increasing their recovery during the precipitation phase.

Supplementary Figure 4: Recovery of [Pyr^1^]apelin-13 from plasma using different extraction methods. Protein precipitation with 80% ACN in water in the presence of half-volume GuHCl (6M) followed by solid phase extraction was used for subsequent experiments.


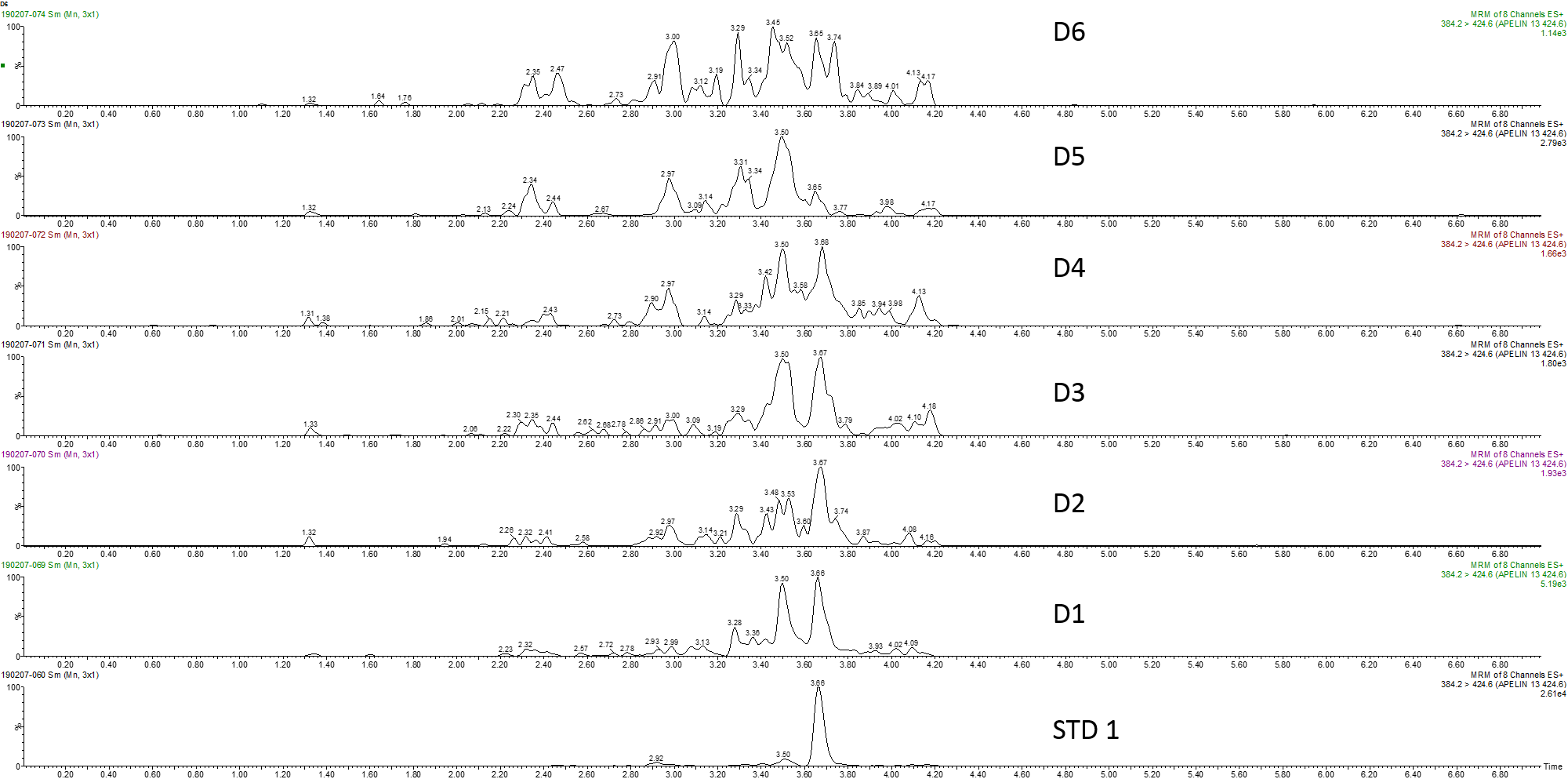


Supplementary Figure 1. Representative chromatograph obtained from the six donor controls that were not infused with [Pyr^1^]apelin-13. STD1, standard 1 (1ng/ml [Pyr^1^]apelin-13); D1-6, donors 1-6.

Supplementary table 1. List of theoretical m/z values of Apelin 13 metabolites with charge states up to 4+.

Where more than one precursor ion was identified, the abundance of the two ions were assessed and compared. Retention times of identified peptides were included.

**References**

1. Read, C. *et al.* Cardiac action of the first G protein biased small molecule apelin agonist. *Biochem. Pharmacol.* **116**, 63–72 (2016).

2. Yang, P. *et al.* Elabela/Toddler Is an Endogenous Agonist of the Apelin APJ Receptor in the Adult Cardiovascular System, and Exogenous Administration of the Peptide Compensates for the Downregulation of Its Expression in Pulmonary Arterial Hypertension. *Circulation* **135**, 1160–1173 (2017).

3. Yang, P. *et al.* A novel cyclic biased agonist of the apelin receptor, MM07, is disease modifying in the rat monocrotaline model of pulmonary arterial hypertension. *Br. J. Pharmacol.* **176**, 1206–1221 (2019).
